# Supplementary figures and images for: Glycogen synthase GYS1 overactivation contributes to glycogen insolubility and malto-oligoglucan-associated neurodegenerative disease
Source: EMBO J. 2025 Jan 13;44(5):1379–413. doi: 10.1038/s44318-024-00339-3 (PMC11876434; doi:10.1038/s44318-024-00339-3)

Blot images corresponding to Fig. 2K

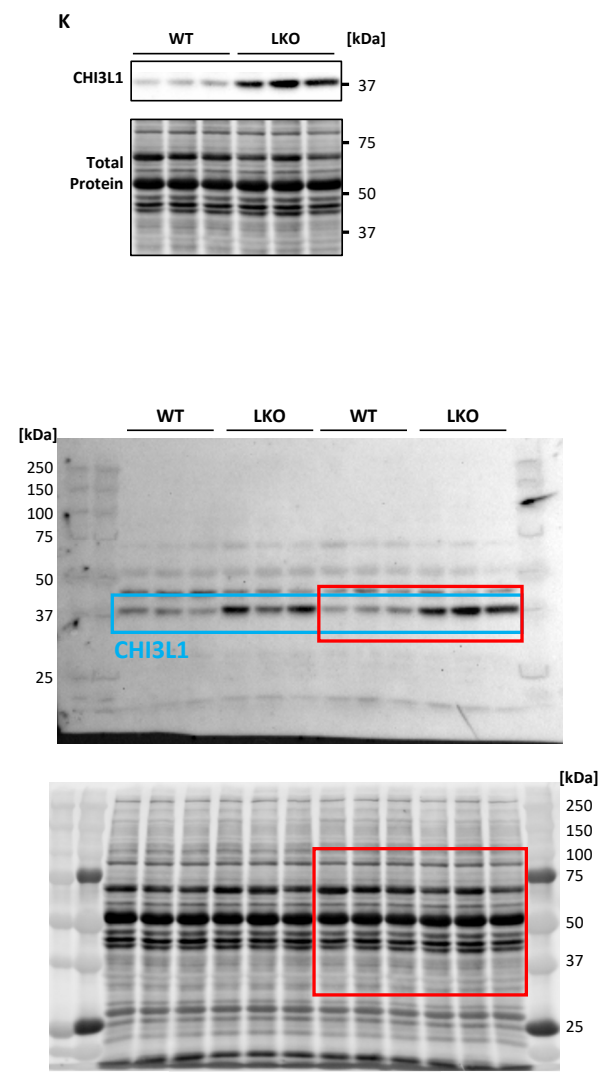

Blot images corresponding to Fig. 2L

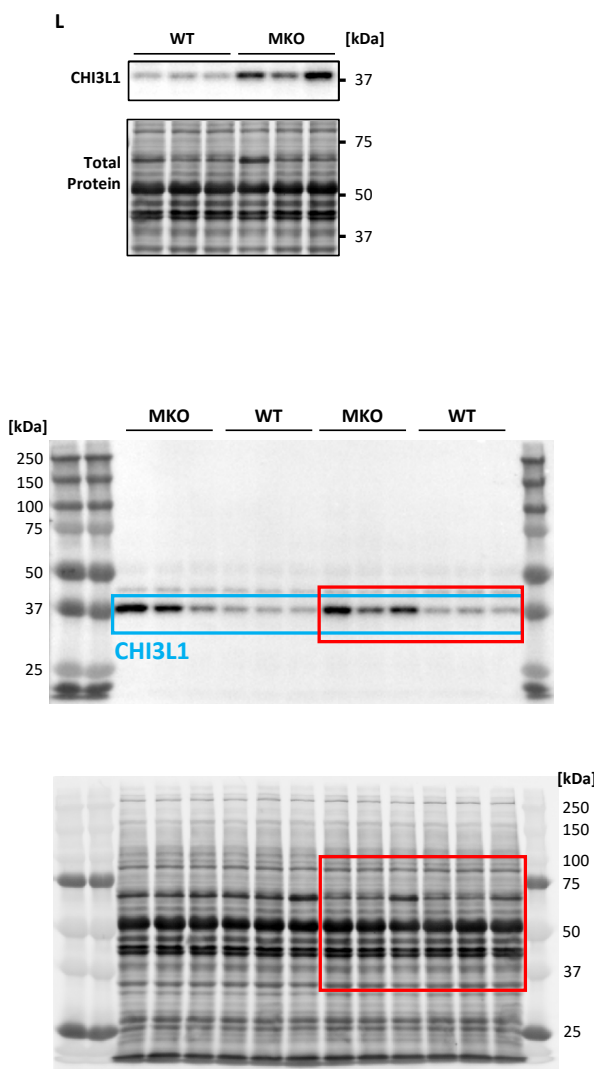

Supplement: Supplementary file 3 — Source data Fig. 2 [file 44318_2024_339_MOESM3_ESM.zip › EMBOJ-2024-117757_SourceDataforFigure2K-L.pdf]

Blot images corresponding to Fig. 5E

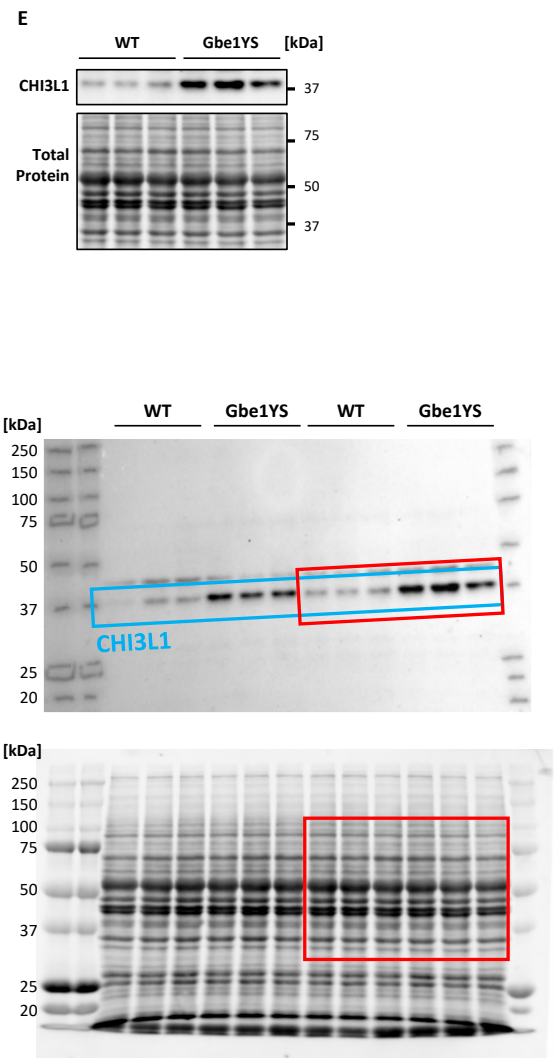

Supplement: Supplementary file 4 — Source data Fig. 5 [file 44318_2024_339_MOESM4_ESM.zip › EMBOJ-2024-117757_SourceDataforFigure5E.pdf]

Blot images corresponding to Fig. 7A

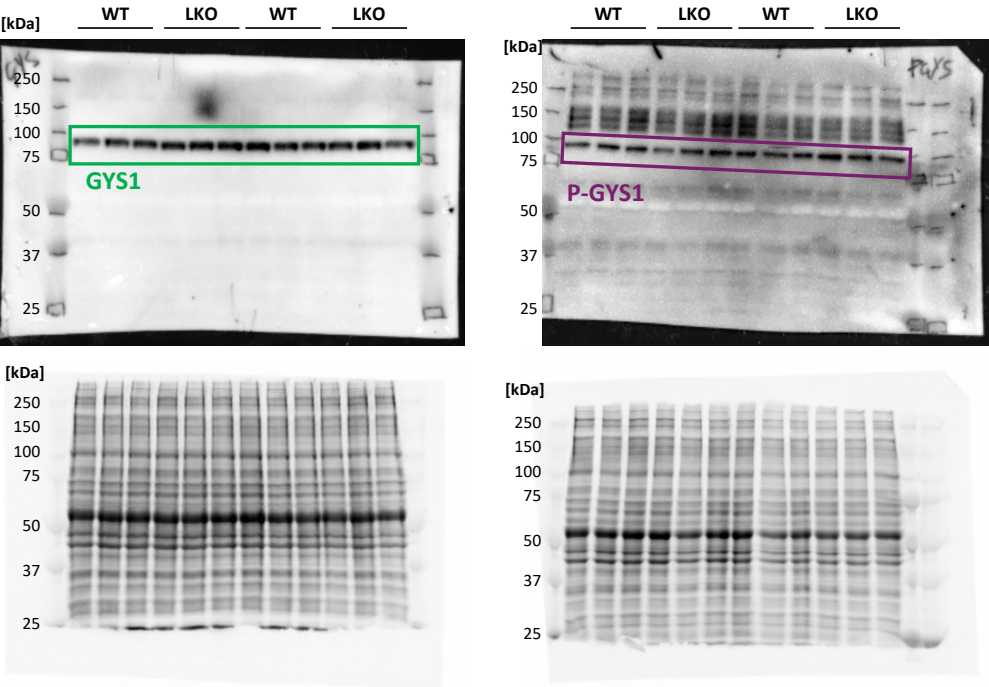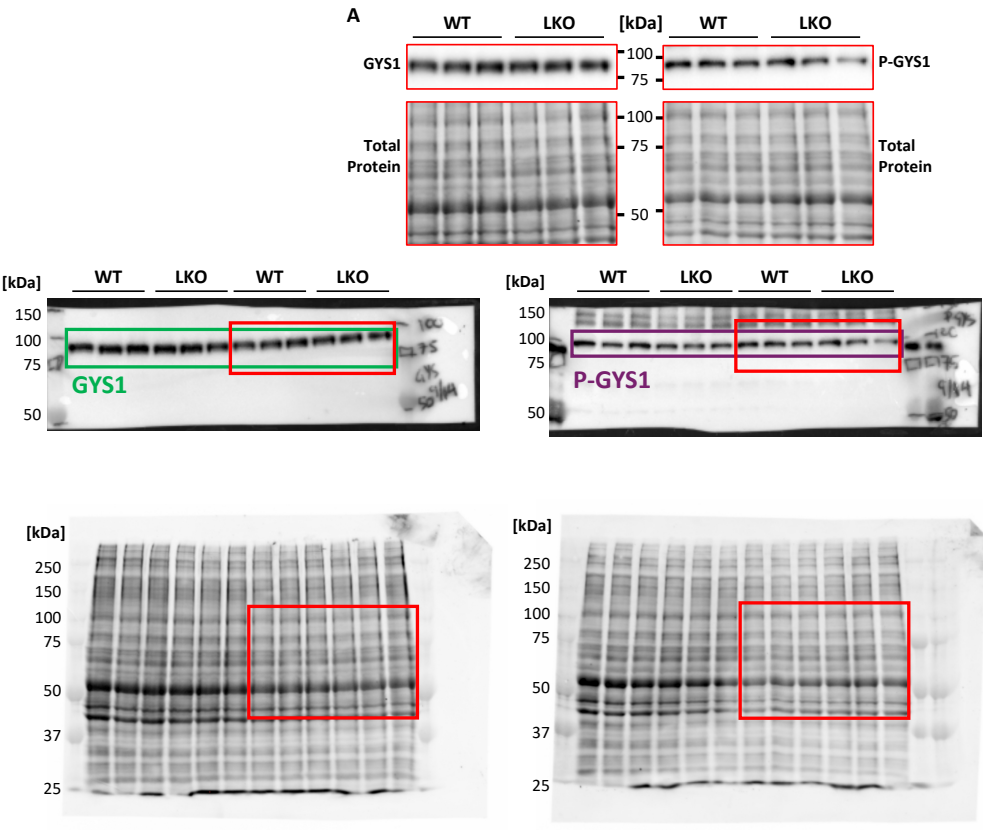

Supplement: Supplementary file 6 — Source data Fig. 7 [file 44318_2024_339_MOESM6_ESM.zip › EMBOJ-2024-117757_SourceDataforFigure7A.pdf]

Blot images corresponding to Fig. 7B

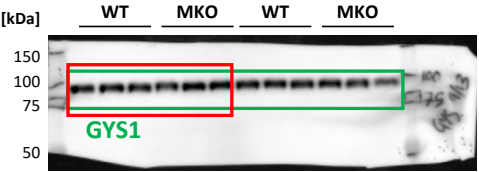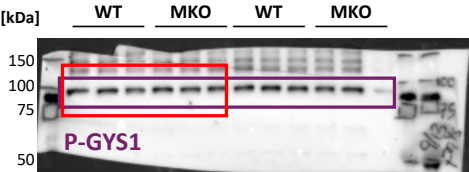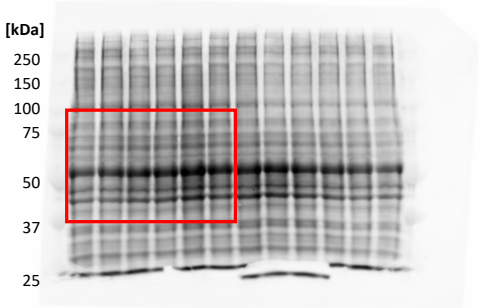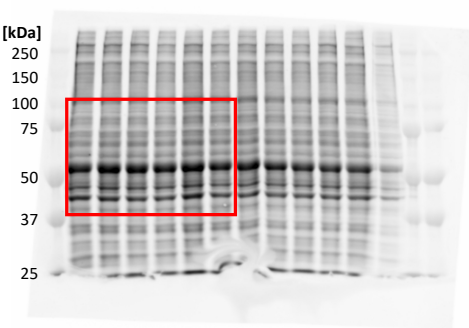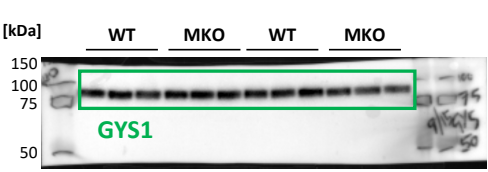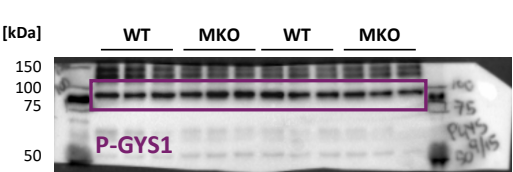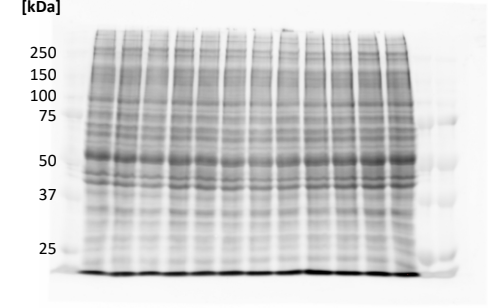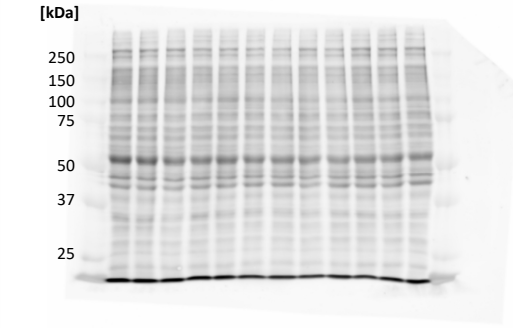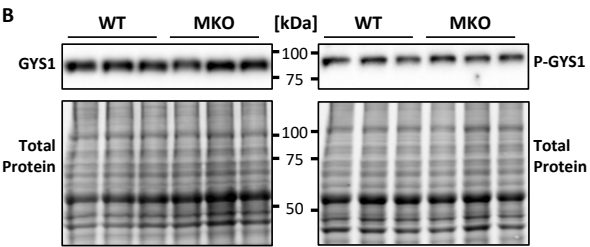

Supplement: Supplementary file 6 — Source data Fig. 7 [file 44318_2024_339_MOESM6_ESM.zip › EMBOJ-2024-117757_SourceDataforFigure7B.pdf]

Blot images corresponding to Fig. 7C

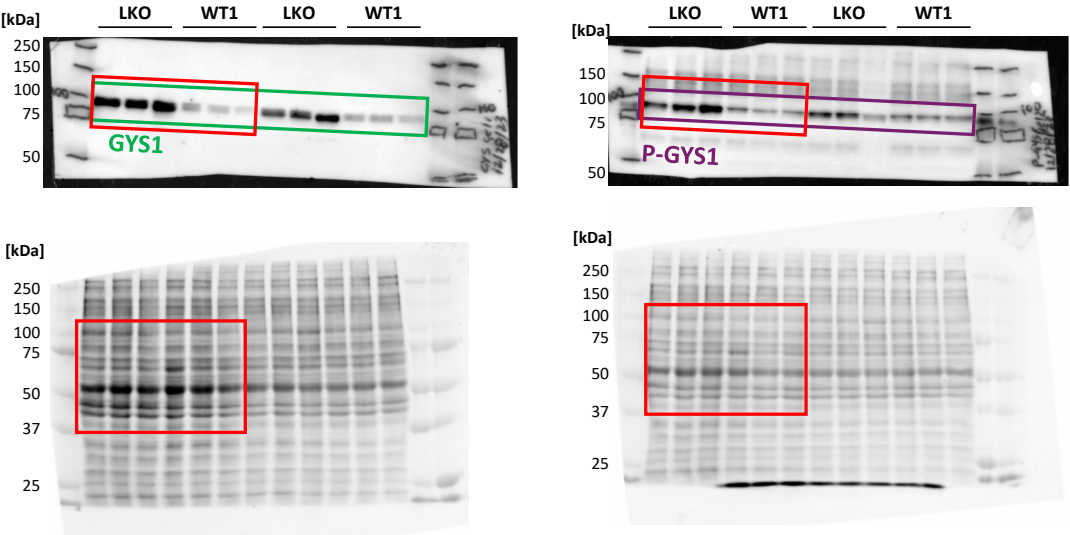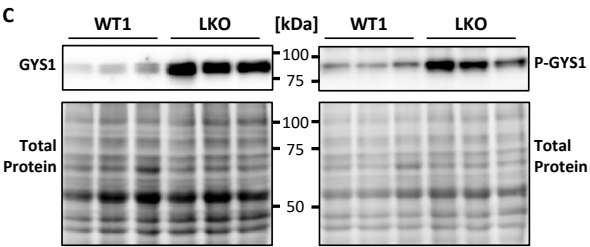

Supplement: Supplementary file 6 — Source data Fig. 7 [file 44318_2024_339_MOESM6_ESM.zip › EMBOJ-2024-117757_SourceDataforFigure7C.pdf]

Blot images corresponding to Fig. 7D

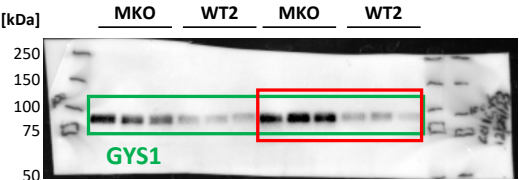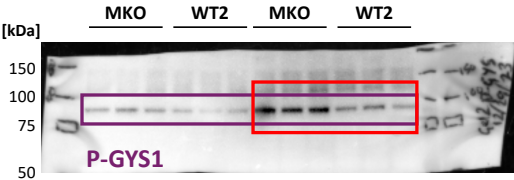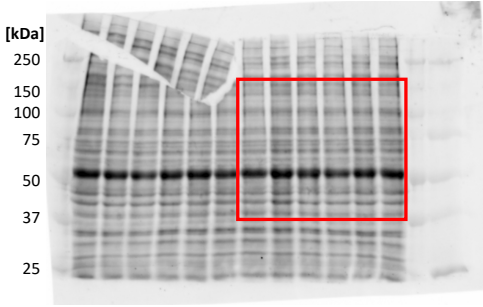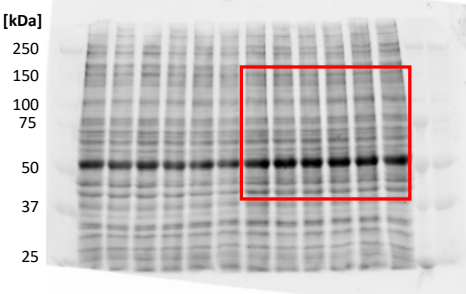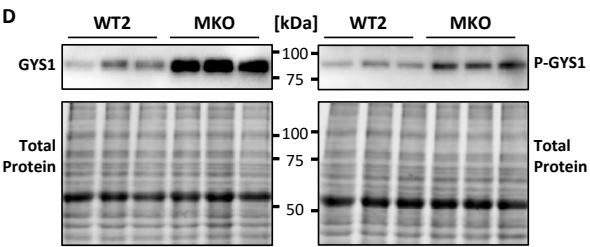

Supplement: Supplementary file 6 — Source data Fig. 7 [file 44318_2024_339_MOESM6_ESM.zip › EMBOJ-2024-117757_SourceDataforFigure7D.pdf]

Blot images corresponding to Fig. 7E

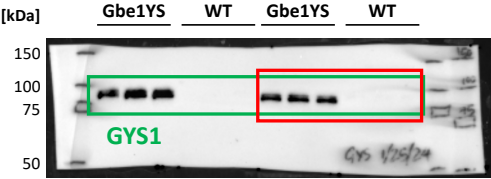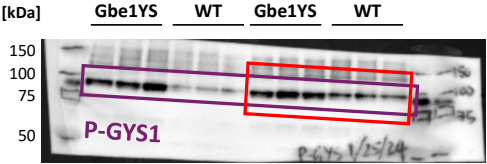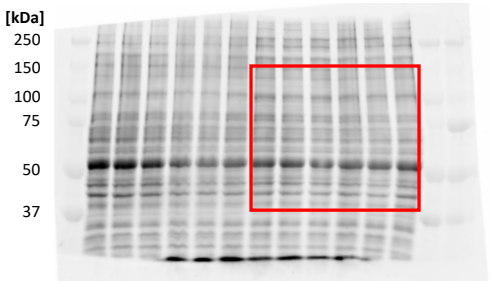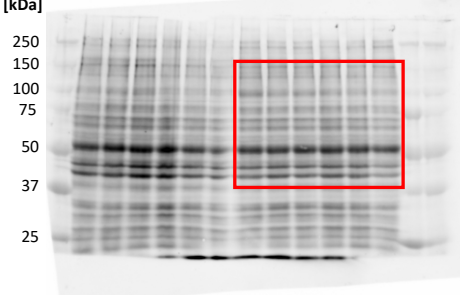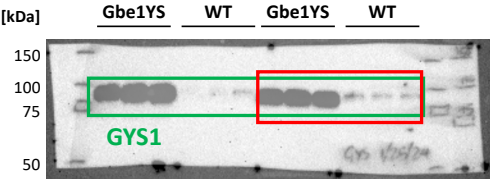

Same blot as above to show presence of GYS1 signal in WTs

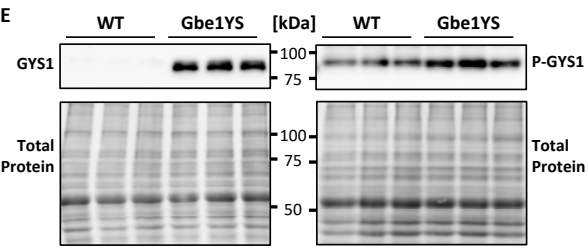

Supplement: Supplementary file 6 — Source data Fig. 7 [file 44318_2024_339_MOESM6_ESM.zip › EMBOJ-2024-117757_SourceDataforFigure7E.pdf]
