# Supplementary material for: Glycogen synthase GYS1 overactivation contributes to glycogen insolubility and malto-oligoglucan-associated neurodegenerative disease
Source: EMBO J. 2025 Jan 13;44(5):1379–413. doi: 10.1038/s44318-024-00339-3 (PMC11876434; doi:10.1038/s44318-024-00339-3)
Supplement: Supplementary file 5 — Source data Fig. 6 [file 44318_2024_339_MOESM5_ESM.zip › EMBOJ-2024-117757_SourceDataforFigure6E,G.pdf]

Blot images corresponding to Fig. 6E

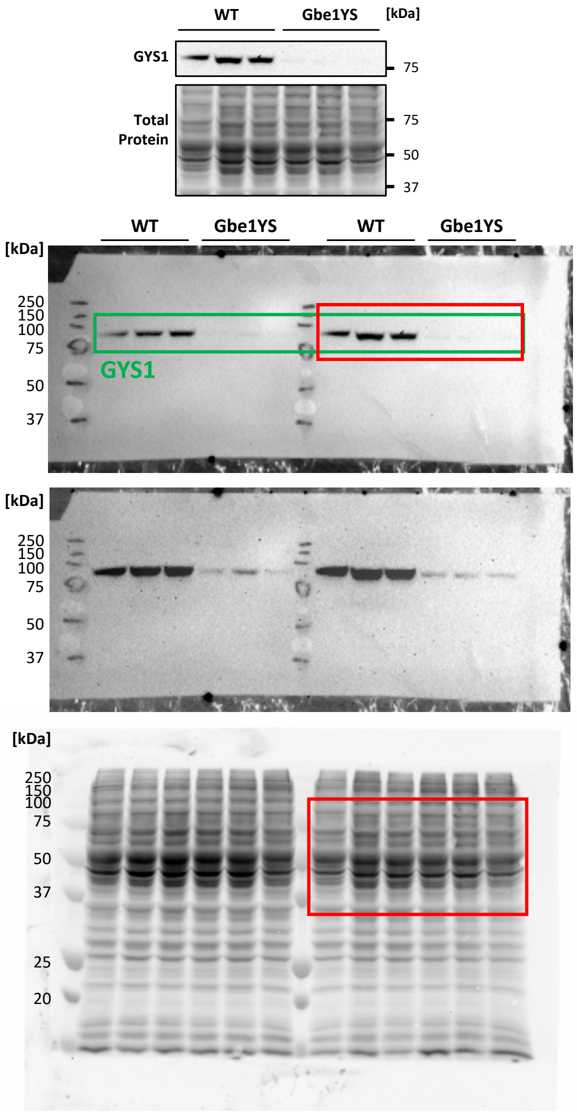

Same blot as above to show presence of GYS1 signal in Gbe1YS

Blot images corresponding to Fig. 6G

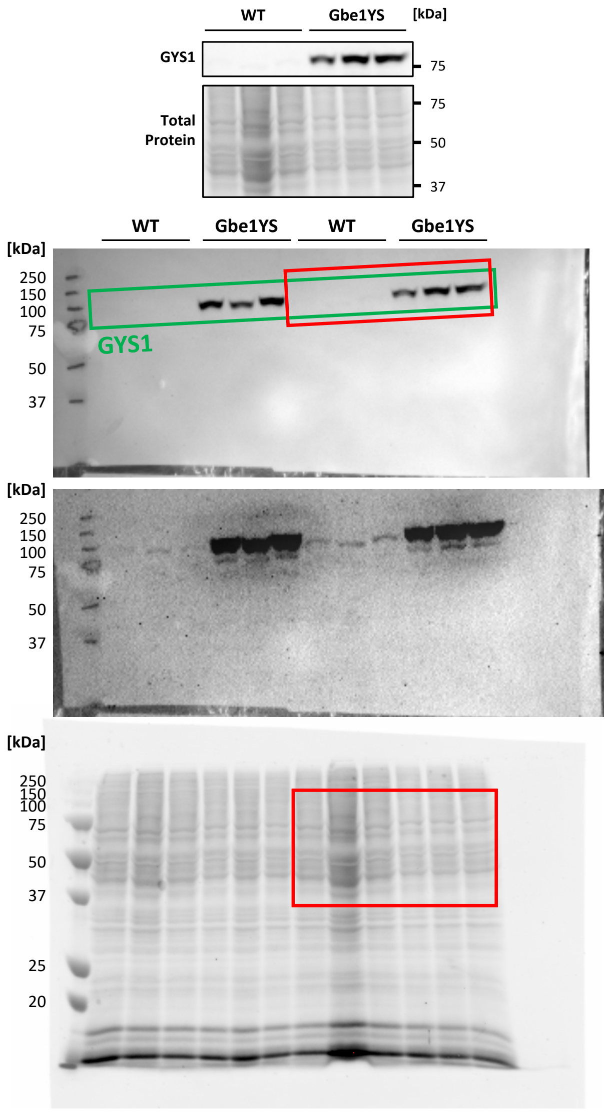

Same blot as above to show presence of GYS1 signal in WT
