# Supplementary material for: Glycogen synthase GYS1 overactivation contributes to glycogen insolubility and malto-oligoglucan-associated neurodegenerative disease
Source: EMBO J. 2025 Jan 13;44(5):1379–413. doi: 10.1038/s44318-024-00339-3 (PMC11876434; doi:10.1038/s44318-024-00339-3)

Hippocampus – CA1

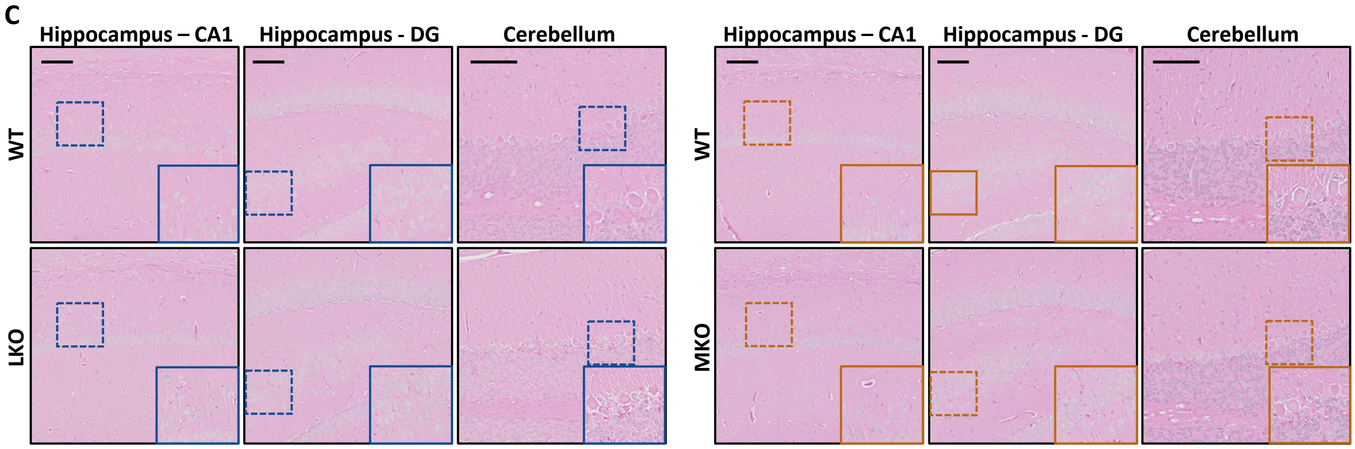

Uncropped images for **Hippocampus – CA1**

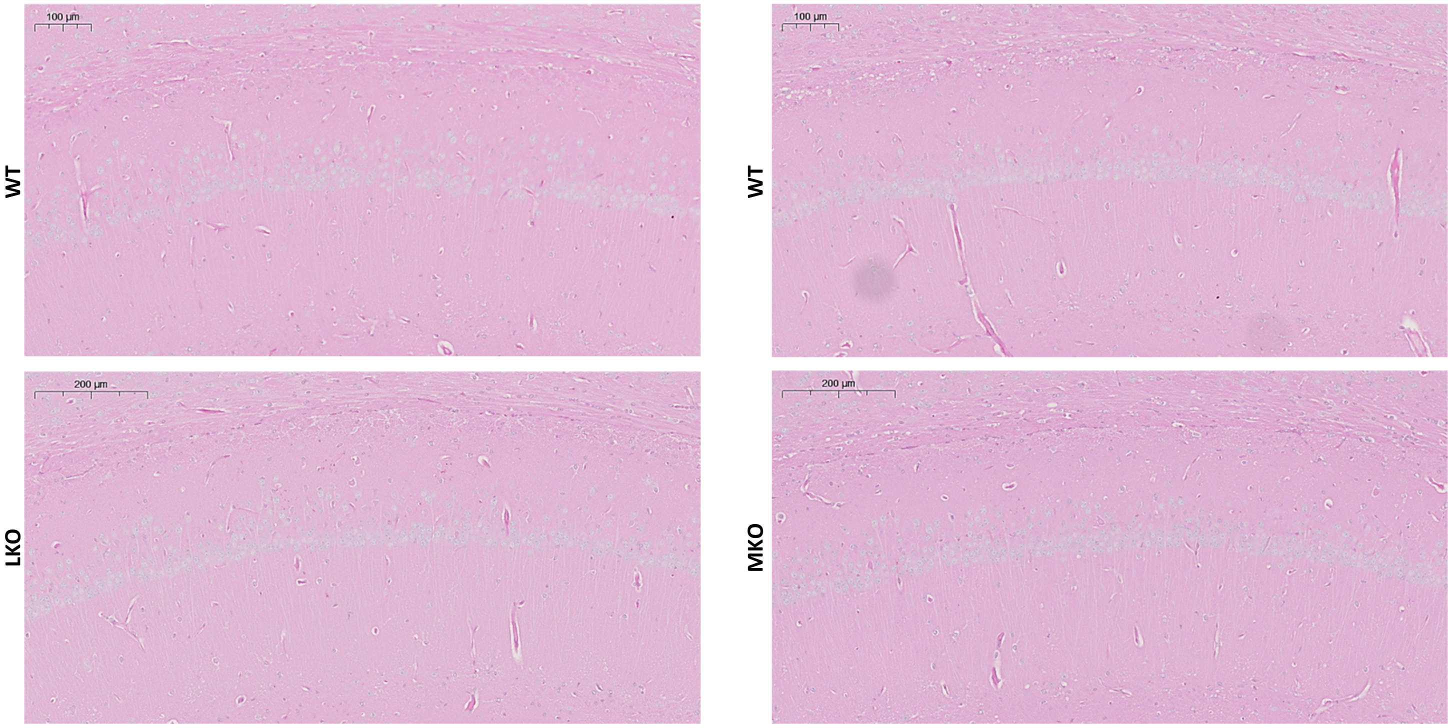

Hippocampus – DG

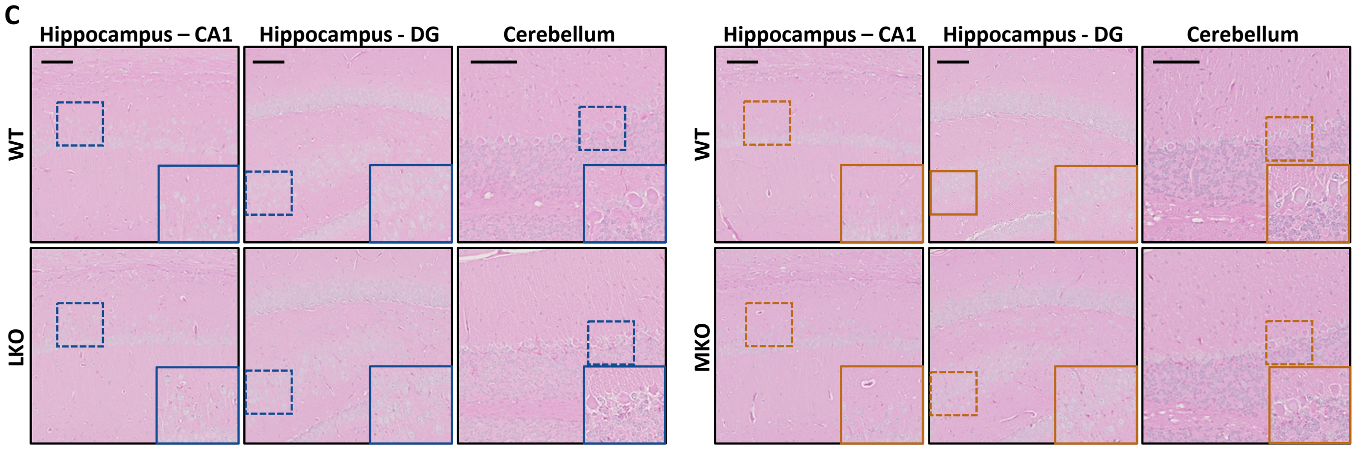

Uncropped images for **Hippocampus – DG**

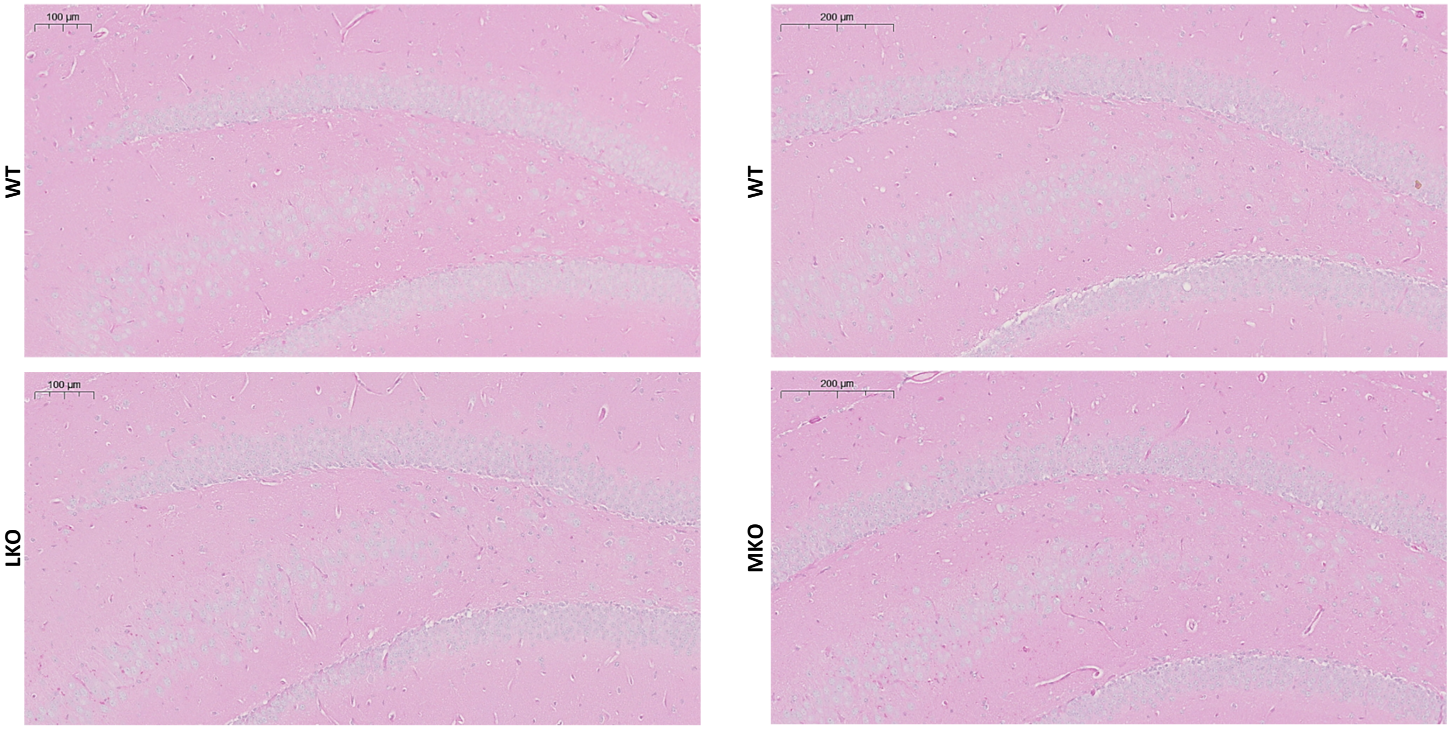

Cerebellum

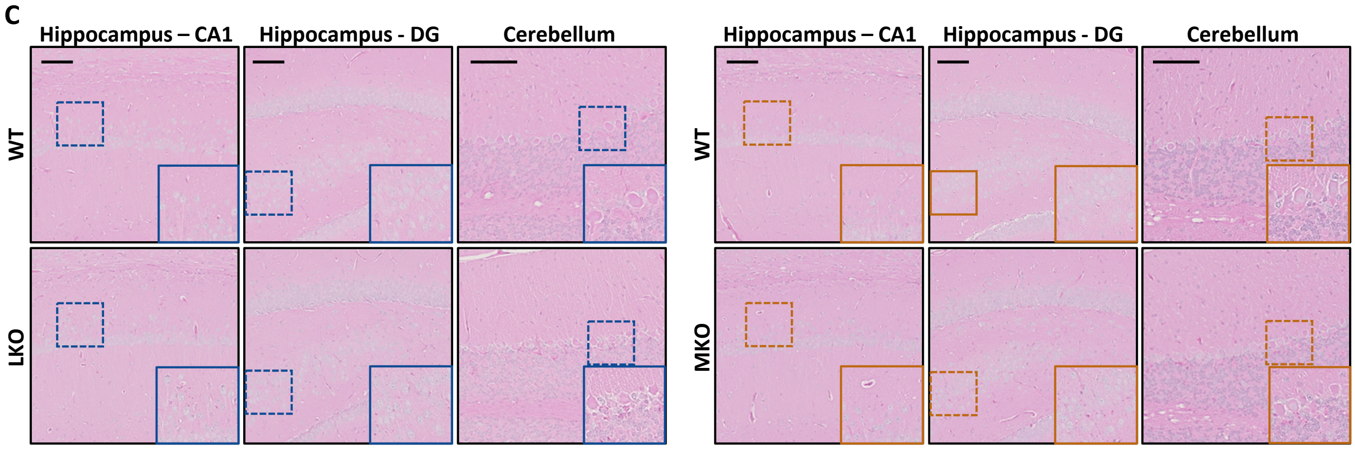

Uncropped images for **Cerebellum**

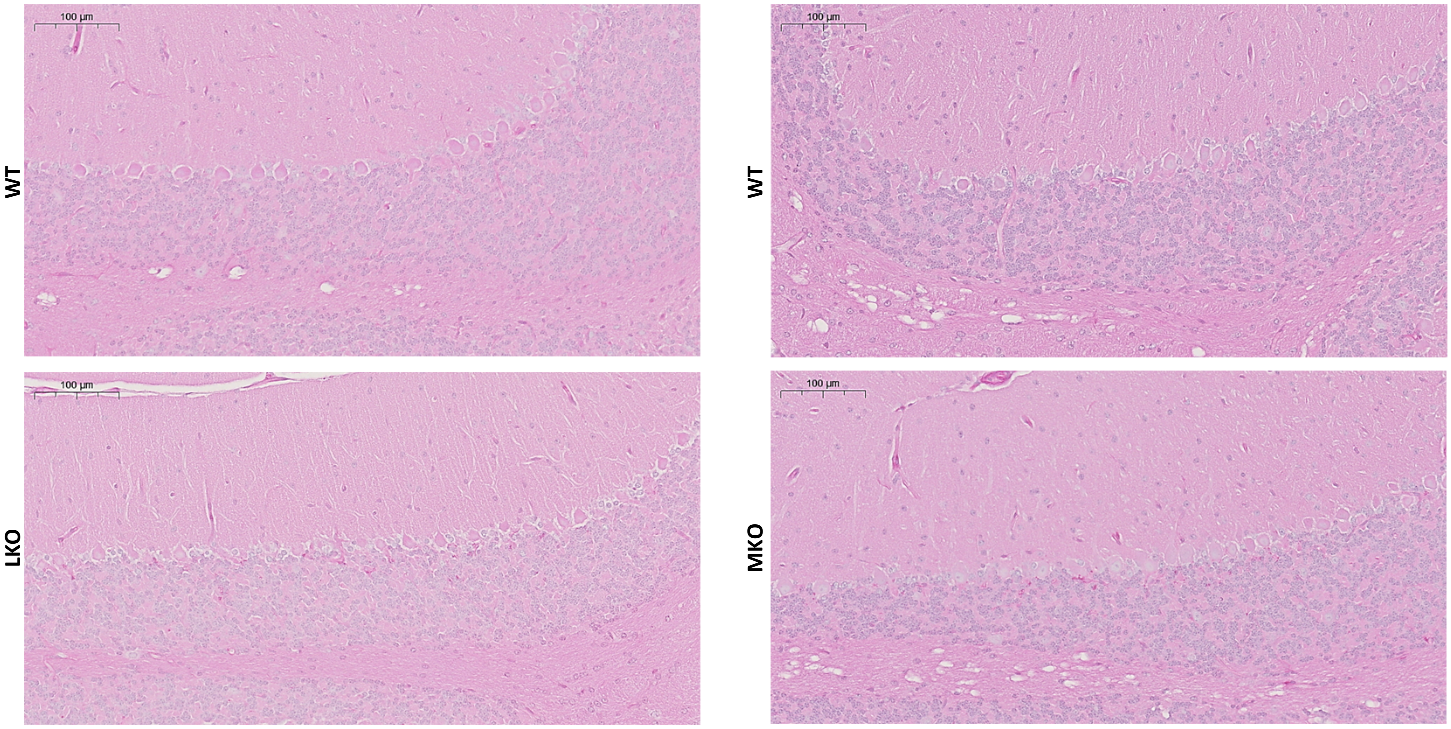

Supplement: Supplementary file 7 — EVFigure Source Data [file 44318_2024_339_MOESM7_ESM.zip › EMBOJ-2024-117757_SourceDataforFigureEV6C.pdf]
