# Supplementary material for: Glycogen synthase GYS1 overactivation contributes to glycogen insolubility and malto-oligoglucan-associated neurodegenerative disease
Source: EMBO J. 2025 Jan 13;44(5):1379–413. doi: 10.1038/s44318-024-00339-3 (PMC11876434; doi:10.1038/s44318-024-00339-3)
Supplement: Supplementary file 8 — Expanded View Figures [file 44318_2024_339_MOESM8_ESM.pdf]

Expanded View Figures

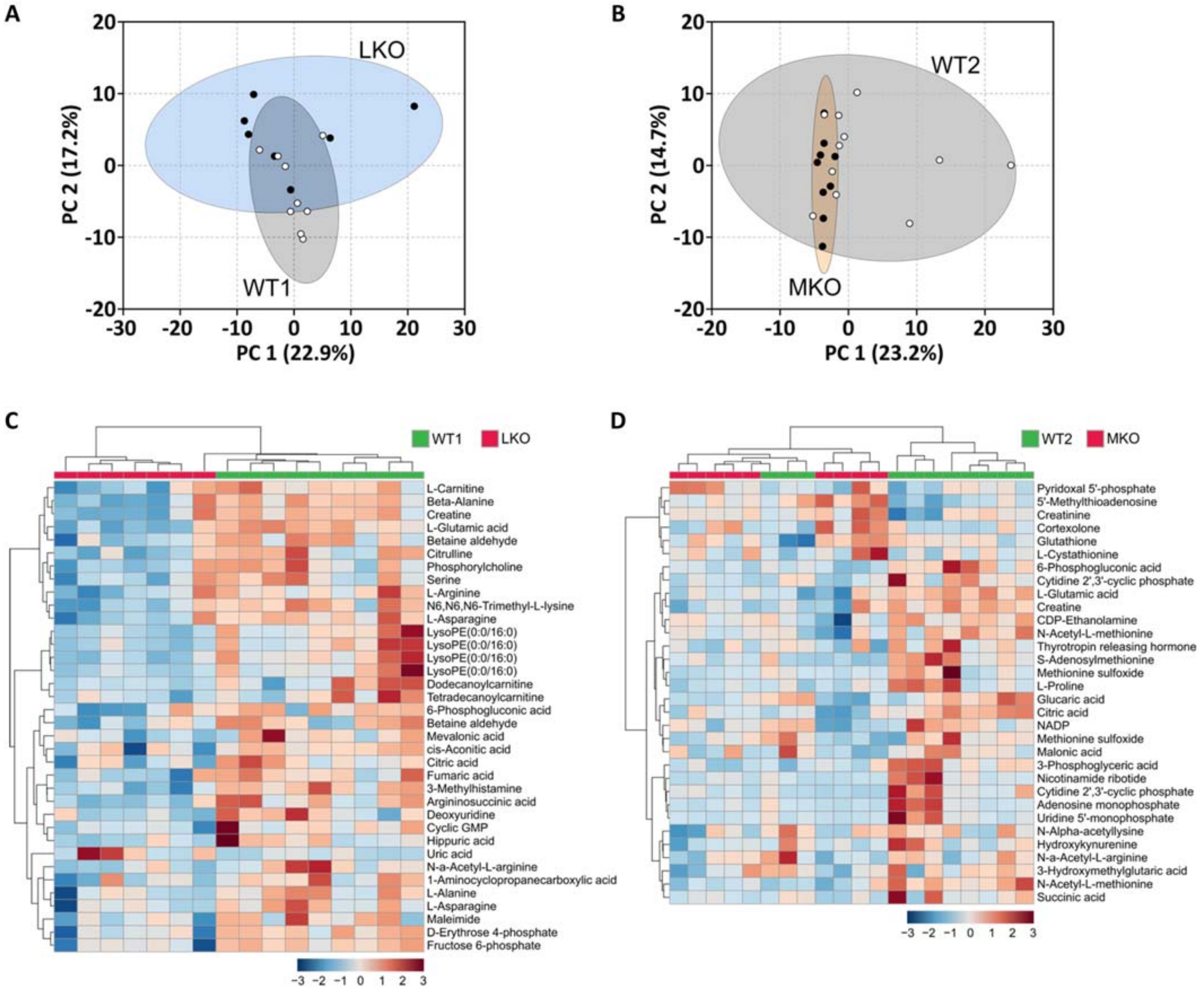

Figure EV1. Cluster analyses using brain metabolite profiles from LD mice showing no or incomplete clustering.

(**A**,**B**) PCA plots of metabolic profiles in LKO (**A**) and MKO (**B**) compared to respective WTs. (**C**, **D**) Heatmaps, comparing all significantly changed ($p < 0.05$) brain metabolites in LKO (**C**) and MKO (**D**) to respective WTs in individual animals from unsupervised Ward cluster analysis with Euclidean distance measure based on unpaired two-tailed *t*-test. Data information: Significance levels determined by MetaboAnalyst 5.0 as follows: $p < 0.05$ (**C**, **D**), $n = 7$–11. Corresponding to Fig. 3. Source data are available online for this figure.

 © The Author(s)

A

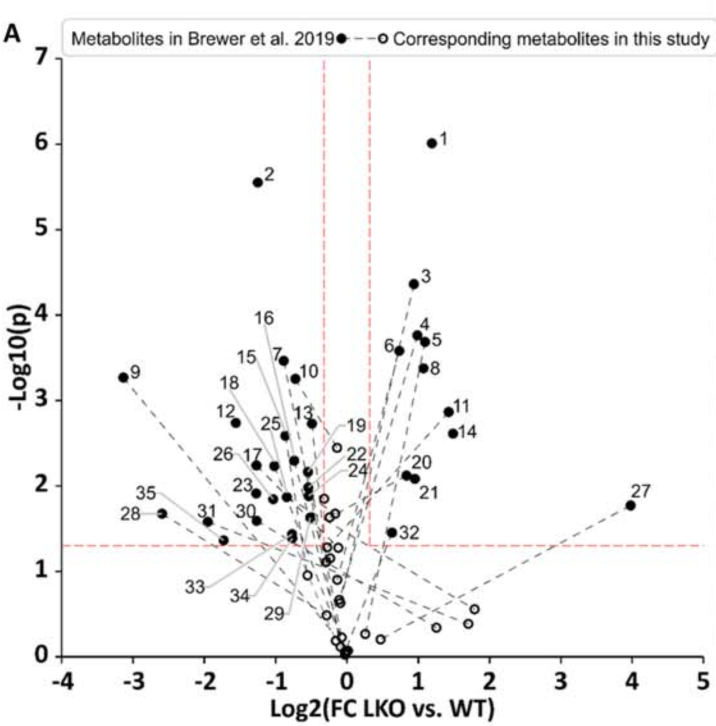

#	Brewer et al. 2019	This study (identified as)
1	Homocysteine	
2	Methyl-nicotinamide	
3	N-Acetyl-aspartic acid	N-Acetyl-L-aspartic acid
4	Lysine	L-Lysine
5	Glycerol-1-phosphate	Glycerol 3-phosphate
6	Taurine	Taurine
7	Deoxy-methylthioadenosine	5'-Methylthioadenosine
8	AXP	
9	Pyruvate	Pyruvic acid
10	Beta-alanine	Beta-Alanine
11	GSSG	Oxidized glutathione
12	Glyceraldehyde	
13	Valine	L-Valine
14	Thymine	
15	Glyceric acid	Glyceric acid
16	Tyrosine	L-Tyrosine
17	N-Acetyl-neuraminic acid	N-Acetylneuraminate
18	N-Acetyl-L-lysine	N-Alpha-acetyllysine
19	Leucine	L-Leucine
20	6-Phosphgluconic acid	6-Phosphogluconic acid
21	Dimethyl-L-lysine monohydrochloride	
22	Fumaric acid	Fumaric acid
23	Ethanolamine	
24	Citrate	Citric acid
25	Uridine	Uridine
26	N-acetyl-galactosamine	
27	DHAP	Dihydroxyacetone phosphate
28	Methylglutamic acid	N-methyl-L-glutamic Acid
29	Malic acid	L-Malic acid
30	Adenosine	Adenosine
31	Guanosine	Guanosine
32	Methionine	Methionine
33	Cysteine	
34	Pyroglutamic acid	Pyroglutamic acid
35	Sucrose	

B

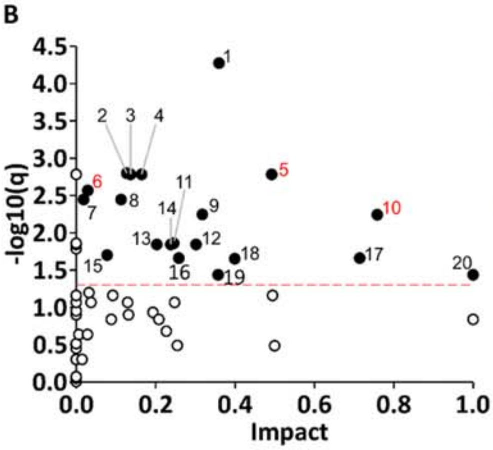

1) Cysteine and methionine metabolism
2) Pyrimidine metabolism
3) Glycerolipid metabolism
4) Tyrosine metabolism
5) **Glyoxylate and dicarboxylate metabolism**
6) **Pantothenate and CoA biosynthesis**
7) Lysine degradation
8) Glycerophospholipid metabolism
9) Glycine, serine and threonine metabolism
10) **Alanine, aspartate and glutamate metabolism**
11) Arginine and proline metabolism
12) Citrate cycle (TCA cycle)
13) Glutathione metabolism
14) Pyruvate metabolism
15) Primary bile acid biosynthesis
16) Glycolysis / Gluconeogenesis
17) Taurine and hypotaurine metabolism
18) beta-Alanine metabolism
19) Phenylalanine metabolism
20) Phenylalanine, tyrosine and tryptophan biosynthesis

C

Duran et al. 2021	Change in MKO	This study (identified as)	MKO vs. WT2 FC	MKO vs. WT2 p	DKO2 vs. WT2 FC	DKO2 vs. WT2 p	
Glycine	down	Glycine	1.04	0.847	0.99	0.975	
Creatinine	up	Creatinine	1.14	0.041 *	1.02	0.824	Rescued in DKO
Ribose-5-phosphate	up	D-Ribose 5-phosphate	0.37	0.151	0.46	0.204	
6-Phosphogluconic acid	up	6-Phosphogluconic acid	0.81	0.003 **	0.81	0.004 **	
Glycolic acid	up	Glycolate	0.60	0.101	0.66	0.212	
Alanine	up	L-Alanine	1.02	0.767	0.92	0.129	
Lactate	up	L-Lactic acid	0.71	0.054	0.78	0.138	
Hexose	up	D-Glucose	1.01	0.925	0.91	0.114	
Lactamide	up	not detected					
Cysteine	up	not detected					

FC 0.33 0.0 3.0

Figure EV2. Meta-analysis of our metabolomic data and published data from CP-fixed LD brains shows no common metabolic signature.

(**A**) Volcano plot of the 35 metabolites significantly changed ($p < 0.05$, FC >1.25 or <0.8) in CP-fixed LKO brains in Brewer et al dataset (Brewer et al, 2019), displayed as filled circles and listed next to the plot. 25 of these metabolites were measured in the current study, are displayed as empty circles, and connected to the corresponding data point from Brewer et al using dashed lines. *P* values were calculated by unpaired two-tailed *t*-test. (**B**) Pathway analysis for published LKO dataset (Brewer et al, 2019). Changed pathways are displayed as filled circles and numbered according to the list next to the plot. Pathways 5, 6, and 10 (red) were also significantly changed in LKO in the current study (see Fig. 3E). (**C**) Table comparing ten metabolites found significantly changed in CP-fixed MKO brains in Duran et al (Duran et al, 2021) with identical metabolites detected in our study with fold changes (FC) and *p* values for MKO or DKO2, respectively, compared to WT2 ($p > 0.05$ or FC of <1.25/>0.8). *P* values were calculated by unpaired two-tailed *t*-test. Data information: Significance levels determined by MetaboAnalyst 5.0 as follows: $p < 0.05$ (**A**, **B**) *$p < 0.05$, **$p < 0.01$ (**C**, red), $n = 7$–11. Corresponding to Fig. 3. Source data are available online for this figure.

 © The Author(s)

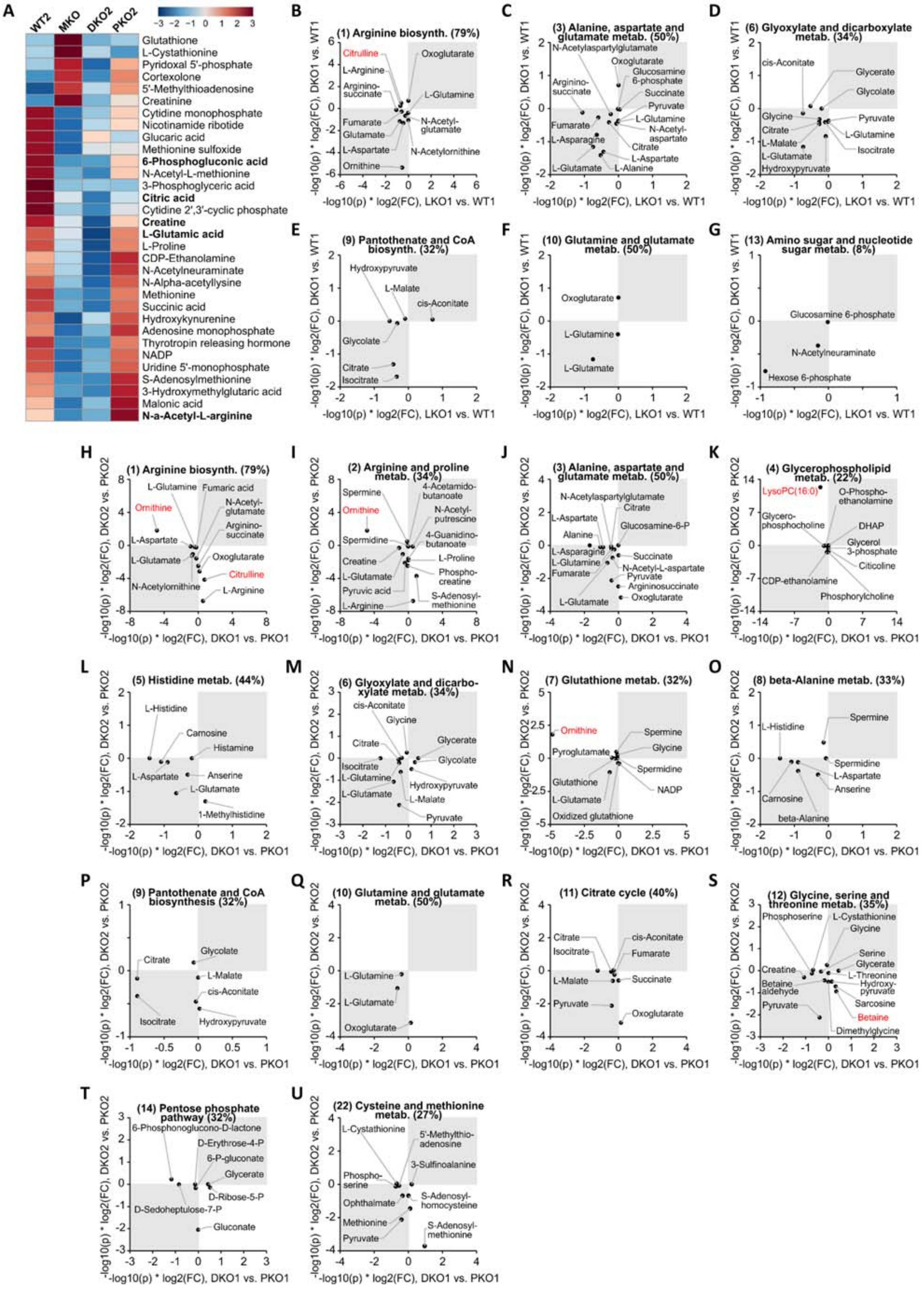
A
WT2
MKO
DKO2
PKO2
−3 −2 −1 0 1 2 3
Glutathione
L-Cystathionine
Pyridoxal 5'-phosphate
Cortexolone
5'-Methylthioadenosine
Creatinine
Cytidine monophosphate
Nicotinamide ribotide
Glucaric acid
Methionine sulfoxide
6-Phosphogluconic acid
N-Acetyl-L-methionine
3-Phosphoglyceric acid
Citric acid
Cytidine 2',3'-cyclic phosphate
Creatine
L-Glutamic acid
L-Proline
CDP-Ethanolamine
N-Acetylneuraminate
N-Alpha-acetyllysine
Methionine
Succinic acid
Hydroxykynurenine
Adenosine monophosphate
Thyrotropin releasing hormone
NADP
Uridine 5'-monophosphate
S-Adenosylmethionine
3-Hydroxymethylglutaric acid
Malonic acid
N-a-Acetyl-L-arginine
B
(1) Arginine biosynth. (79%)
Citrulline
L-Arginine
Arginino-succinate
Fumarate
Glutamate
L-Aspartate
Ornithine
Oxoglutarate
L-Glutamine
N-Acetyl-glutamate
N-Acetylornithine
C
(3) Alanine, aspartate and glutamate metab. (50%)
N-Acetylaspartylglutamate
Oxoglutarate
Glucosamine 6-phosphate
Succinate
Pyruvate
L-Glutamine
N-Acetyl-aspartate
Citrate
L-Aspartate
L-Alanine
Arginino-succinate
Fumarate
L-Asparagine
L-Glutamate
D
(6) Glyoxylate and dicarboxylate metab. (34%)
cis-Aconitate
Glycerate
Glycolate
Pyruvate
L-Glutamine
Isocitrate
Glycine
Citrate
L-Malate
L-Glutamate
Hydroxypyruvate
E
(9) Pantothenate and CoA biosynth. (32%)
Hydroxypyruvate
L-Malate
cis-Aconitate
Glycolate
Citrate
Isocitrate
F
(10) Glutamine and glutamate metab. (50%)
Oxoglutarate
L-Glutamine
L-Glutamate
G
(13) Amino sugar and nucleotide sugar metab. (8%)
Glucosamine 6-phosphate
N-Acetylneuraminate
Hexose 6-phosphate
-log10(p) * log2(FC), LKO1 vs. WT1
-log10(p) * log2(FC), DKO1 vs. WT1
H
(1) Arginine biosynth. (79%)
L-Glutamine
Fumaric acid
Ornithine
N-Acetyl-glutamate
Arginino-succinate
L-Aspartate
L-Glutamate
Oxoglutarate
Citrulline
N-Acetylornithine
L-Arginine
I
(2) Arginine and proline metab. (34%)
Spermine
4-Acetamido-butanoate
Ornithine
N-Acetyl-putrescine
Spermidine
4-Guanidino-butanoate
Creatine
L-Proline
L-Glutamate
Phospho-creatine
Pyruvic acid
S-Adenosyl-methionine
L-Arginine
J
(3) Alanine, aspartate and glutamate metab. (50%)
N-Acetylaspartylglutamate
Citrate
L-Aspartate
Glucosamine-6-P
Alanine
L-Asparagine
L-Glutamine
Fumarate
Succinate
N-Acetyl-L-aspartate
Pyruvate
Argininosuccinate
Oxoglutarate
L-Glutamate
K
(4) Glycerophospholipid metab. (22%)
LysoPC(16:0)
O-Phospho-ethanolamine
Glycero-phosphocholine
DHAP
Glycerol 3-phosphate
Citicoline
CDP-ethanolamine
Phosphorylcholine
L
(5) Histidine metab. (44%)
L-Histidine
Carnosine
Histamine
L-Aspartate
Anserine
L-Glutamate
1-Methylhistidine
M
(6) Glyoxylate and dicarboxylate metab. (34%)
cis-Aconitate
Glycine
Citrate
Glycerate
Glycolate
Isocitrate
L-Glutamine
L-Glutamate
Hydroxypyruvate
L-Malate
Pyruvate
N
(7) Glutathione metab. (32%)
Ornithine
Spermine
Glycine
Pyroglutamate
Spermidine
Glutathione
NADP
L-Glutamate
Oxidized glutathione
O
(8) beta-Alanine metab. (33%)
L-Histidine
Spermine
Spermidine
L-Aspartate
Anserine
Carnosine
beta-Alanine
P
(9) Pantothenate and CoA biosynthesis (32%)
Citrate
Glycolate
L-Malate
cis-Aconitate
Isocitrate
Hydroxypyruvate
Q
(10) Glutamine and glutamate metab. (50%)
L-Glutamine
L-Glutamate
Oxoglutarate
R
(11) Citrate cycle (40%)
Citrate
cis-Aconitate
Isocitrate
Fumarate
L-Malate
Succinate
Pyruvate
Oxoglutarate
S
(12) Glycine, serine and threonine metab. (35%)
Phosphoserine
L-Cystathionine
Glycine
Serine
Glycerate
Creatine
L-Threonine
Betaine aldehyde
Hydroxy-pyruvate
Pyruvate
Sarcosine
Betaine
Dimethylglycine
T
(14) Pentose phosphate pathway (32%)
6-Phosphonoglucono-D-lactone
D-Erythrose-4-P
6-P-gluconate
Glycerate
D-Ribose-5-P
D-Sedoheptulose-7-P
Gluconate
U
(22) Cysteine and methionine metab. (27%)
L-Cystathionine
5'-Methylthio-adenosine
3-Sulfinoalanine
Phospho-serine
S-Adenosyl-homocysteine
Ophthalmate
Methionine
Pyruvate
S-Adenosyl-methionine
-log10(p) * log2(FC), DKO1 vs. PKO1
-log10(p) * log2(FC), DKO2 vs. PKO2

Figure EV3. Metabolite and pathway changes in LKO and MKO not rescued by PTG knockout.

(**A**) Heatmap for malin cohort, comparing all significantly changed metabolites in MKO between the four genotypes. Metabolites significantly changed in both LD mice in bold. (**B–G**) Volcano plot areas of all detected metabolites from pathways #1 (**B**), #3 (**C**), #6 (**D**), #9 (**E**), #10 (**F**), and #13 (**G**) used for calculation of mean areas in Fig. 4J plotted for LKO vs. WT1 comparison with DKO1 vs. WT1. *P* values for each metabolite were calculated by unpaired two-tailed *t*-test. (**H–U**) Volcano plot areas of all detected metabolites from pathways #1 (**H**), #2 (**I**), #3 (**J**), #4 (**K**), #5 (**L**), #6 (**M**), #7 (**N**), #8 (**O**), #9 (**P**), #10 (**Q**), #11 (**R**), #12 (**S**), #14 (**T**), and #22 (**U**) used for calculation of mean areas in Fig. 4K plotted for DKO1 vs. PKO1 comparison with DKO2 vs. PKO2. *P* values for each metabolite were calculated by unpaired two-tailed *t*-tests. Data information: Off-centered dots in **B–U** correspond to metabolites with increased fold-change (FC) and/or significance. Red-font metabolites were significantly changed in both genotype comparisons but with different directionality. Pathway numbers as in Fig. 4A. Corresponding to Fig. 4. Percentages indicate detected metabolites of all metabolites in the pathway. Source data are available online for this figure.

 © The Author(s)

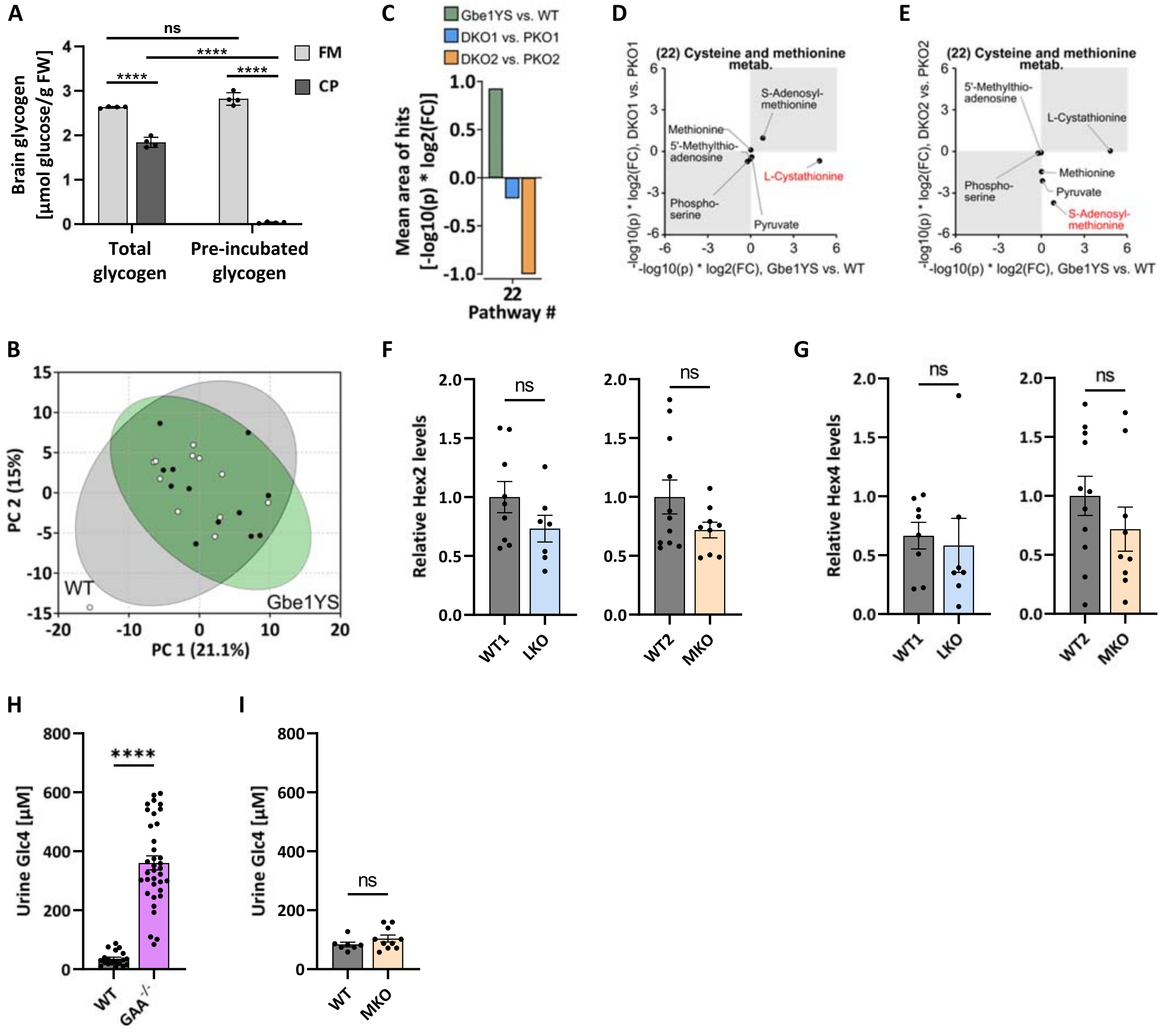

Figure EV4. Increased Hex4/Glc4 levels in Gbe1YS, but not LD mice are comparable to changes in Pompe disease.

(**A**) Brain total and preincubated glycogen in CP- and FM-fixed brains of WT mice from Gbe1YS cohort. $n = 4$. (**B**) PCA plot of metabolic profile in Gbe1YS compared to respective WT. $n = 12$. (**C**) The mean area of hits to show directionality of change (calculations as shown in Fig. 4I) displayed for pathway #22 that was significantly changed in all three indicated genotype comparisons. *P* values for each metabolite in the pathway were calculated by unpaired two-tailed *t*-tests. (**D**, **E**) Volcano plot areas of metabolites from pathway #22 used for calculation of mean areas in G plotted for Gbe1YS ($n = 12$) vs. WT ($n = 12$) comparison with DKO1 ($n = 10$) vs. PKO1 ($n = 8$) (**D**) or DKO2 ($n = 10$) vs. PKO2 ($n = 6$) (**E**), respectively. Off-centered dots correspond to metabolites with increased fold-change (FC) and/or significance. Red-font metabolites were significantly changed in both genotype comparisons but with different directionality. *P* values for each metabolite were calculated by unpaired two-tailed *t*-tests. (**F**, **G**) Relative metabolite levels of hexose disaccharide Hex2 (**F**) and hexose tetrasaccharide Hex4 (**G**) in LKO and MKO mice compared to their respective WTs. $n = 7$–11. (**H**, **I**) Urine Glc4 levels in Pompe disease mice $GAA^{-/-}$ (**H**) and MKO mice (**I**). $n = 19$–36 (**H**), $n = 7$–10 (**I**). Data information: Data in (**A**) and (**F–I**) are presented as mean ± SEM. ****$p < 0.0001$ by two-way ANOVA with Tukey post hoc analysis (**A**) or by Welch's *t*-test (**F–I**); ns not significant, CP cryopreservation, FM focused microwave. Corresponding to Fig. 5. Source data are available online for this figure. *P* values: (**A**) Tot. (FM vs. CP) 2.2E-07, Pre. (FM vs. CP) 2.4E-13, CP (Tot. vs. Pre.) 7.6E-12, FM (Tot. vs. Pre.) 0.058; (**H**) <1.0E-15. Source data are available online for this figure.

© The Author(s)

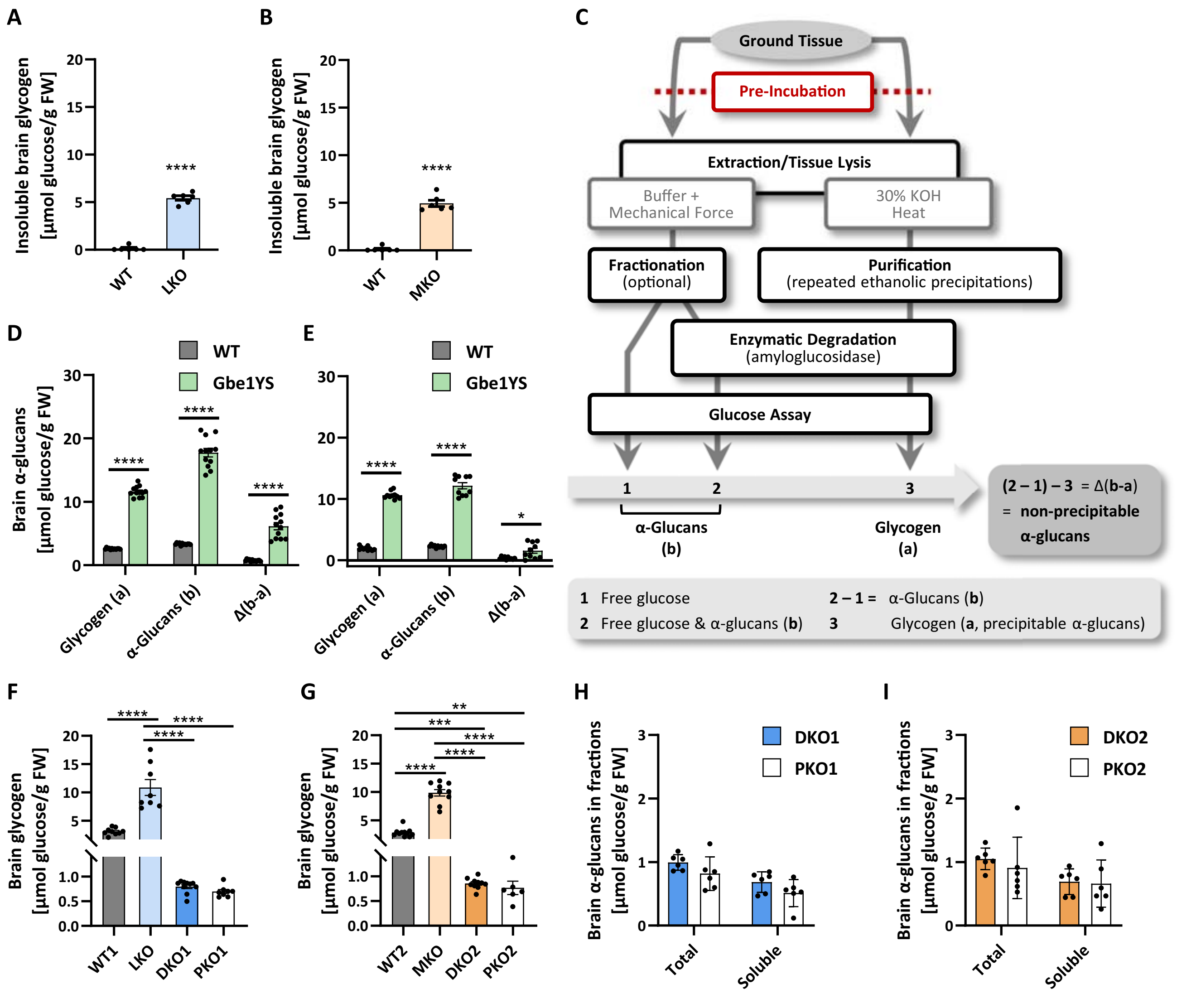

Figure EV5. Metabolically volatile α-glucan is present in Gbe1YS but absent in DKO mice showing rescue compared to LD mice.

(**A**, **B**) Insoluble (preincubated) glycogen in LKO (**A**) and MKO (**B**) mice after CP fixation. $n = 6$. (**C**) Schematic showing methodology of glycogen determination as precipitable glycogen (a) or α-glucan (b) which additionally contains lower molecular weight glucans (≥2 glucose units). (**D**, **E**) Brain α-glucans in Gbe1YS mice after FM (**D**) or CP (**E**) fixation. Terminology is explained in (**C**). $n = 10$–12. (**F**, **G**) Brain total glycogen following FM fixation in laforin (**F**) and malin (**G**) cohort as in Fig. 1C but with different y-axis scale. $n = 6$–11. (**H**, **I**) Total and soluble brain α-glucan in PKO and DKO from the laforin (**H**) and malin (**I**) cohort after FM fixation. $n = 6$. Data information: All data were presented as mean ± SEM. $*p < 0.05$; $**p < 0.01$; $***p < 0.001$; $****p < 0.0001$ by two-way ANOVA with Tukey post hoc analysis (**F**, **G**) or by Welch's t-test (**A**–**E**, **H**, **I**). Corresponding to Fig. 6. P values: (**A**) 1.7E-07; (**B**) 1.03E-05; (**D**) Glyc. 3.4E-13, α-Gluc. 2.6E-10, Δ(a-b) 9.7E-07; (**E**) Glyc. <1.0E-15, α-Gluc. 7.5E-09, Δ(a-b) 0.0147; (**F**) WT1 vs. LKO 1.3E-08, LKO vs. DKO1 1.7E-11, LKO vs. PKO1 4.9E-11; (**G**) WT2 vs. MKO 5.6E-14, MKO vs. DKO2 5.5E-14, MKO vs. PKO2 5.5E-14, WT2 vs. DKO2 0.00063, WT2 vs. PKO2 0.0022. Source data are available online for this figure.

© The Author(s)

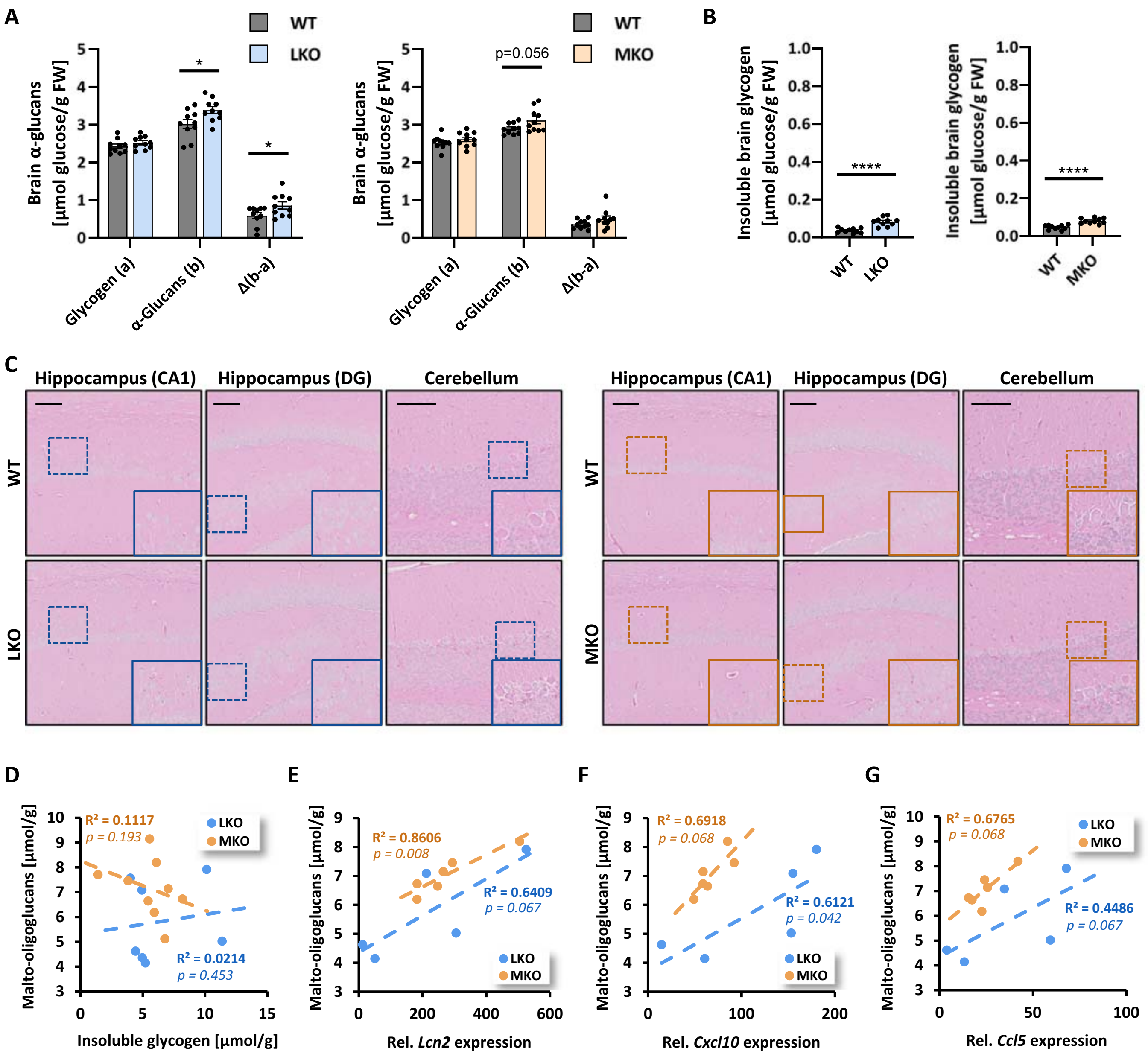

Figure EV6. Malto-oligoglucan accumulation only found in aged LD mice correlates well with neuroinflammatory marker expression.

(**A**) Brain α-glucans in 5-week-old LKO and MKO, respectively, compared to the respective WT after FM fixation. Terminology explained in Fig. EV5C. $n = 10$. (**B**) Insoluble (preincubated) glycogen in 5-week-old LKO and MKO mice, respectively after CP fixation. $n = 10$. (**C**) Representative PASD images of hippocampus and cerebellum in LKO and MKO mice, respectively. Scale bar, 100 µm. The region highlighted by small square is shown enlarged at the right bottom of each image (bigger squares outlined in blue or orange). (**D–G**) Correlation coefficients (R^2) between malto-oligoglucans and insoluble glycogen (**D**) or neuroinflammatory markers *Lcn2* (**E**), *Cxcl10* (**F**), and *Ccl5* (**G**), respectively, in aged LKO and MKO. $n = 5$–9. Data information: Data were presented as mean ± SEM (**A**, **B**) or individual data points (**D–G**). $^{*}p < 0.05$; $^{****}p < 0.0001$ by Welch's *t*-test. Corresponding to Fig. 6. *P* values: (**A**) α-Gluc.0.0265, Δ(a-b) 0.0439; (**B**) WT vs. LKO 5.0E-05, WT vs. MKO 2.5E-05. Source data are available online for this figure.
